# Post-transplant cyclophosphamide for graft-versus-host disease prophylaxis in HLA matched sibling or matched unrelated donor transplant for patients with acute leukemia, on behalf of ALWP-EBMT

**DOI:** 10.1186/s13045-018-0586-4

**Published:** 2018-03-15

**Authors:** Annalisa Ruggeri, Myriam Labopin, Andrea Bacigalupo, Boris Afanasyev, Jan J. Cornelissen, Ahmet Elmaagacli, Maija Itälä-Remes, Didier Blaise, Ellen Meijer, Yener Koc, Noel Milpied, Harry C. Schouten, Nicolaus Kroeger, Mohamad Mohty, Arnon Nagler

**Affiliations:** 1 Service d’Hématologie et Thérapie Cellulaire, Hôpital Saint Antoine, AP-HP, 168 Rue du Faubourg Saint Antoine, 75012 Paris, France; 2 Department of Pediatric Hematology and Oncology, IRCCS Bambino Gesù Children’s Hospital, Piazza SOnofrio, 4, Roma, 00165 Italy; 3 INSERM, UMRs 938, Paris, France; 4 Department of Haematology, Università Cattolica del Sacro Cuore, Rome, Italy; 5 Raisa Gorbacheva Memorial Research Institute for Paediatric Oncology, Hematology and Transplantation, St. Petersburg, Russia; 6 Department of Hematology, Erasmus MC Cancer Institute, University Medical Center Rotterdam, Rotterdam, Netherlands; 7 Asklepios Klinik St. George, Lohmühlenstrasse, Hamburg, Germany; 8 Stem Cell Transplant Unit, Turku University Hospital, Turku, Finland; 9 Transplantation and Therapie Cellulaire, Institut Paoli Calmettes, Marseille, France; 10 Department of Hematology, University Medical Center, Amsterdam, Netherlands; 11 Stem Cell Transplant Unit, Medical Park Hospitals, Antalya, Turkey; 12 CHU Bordeaux, Hôpital Haut-leveque, Pessac, France; 13 Department of Internal Medicine, Section of Hematology, University Hospital Maastricht, Maastricht, Netherlands; 14 Department of Stem cell Transplantation, University Hospital Eppendorf, Hamburg, Germany; 15 Université Pierre et Marie Curie, Paris, France; 16 Division of Hematology and Bone Marrow Transplantation, The Chaim Sheba Medical Center, Tel-Hashomer, Tel Aviv University (TAU), Tel Aviv, Israel

**Keywords:** Hematopoietic stem cell transplantation, Post-transplantation cyclophosphamide, Stem cell source, Acute leukemia, Acute graft-versus-host disease

## Abstract

**Background:**

Experience using post-transplant cyclophosphamide (PT-Cy) as graft-versus-host disease (GVHD) prophylaxis in allogeneic stem cell transplantation (HSCT) from matched sibling donors (MSD) or unrelated donors (UD) is limited and with controversial results. The study aim was to evaluate PT-Cy as GVHD prophylaxis post-HSCT from MSD and UD transplants. We analyzed 423 patients with acute leukemia who received PT-Cy alone or in combination with other immunosuppressive (IS) drugs as GVHD prophylaxis. Seventy-eight patients received PT-Cy alone (group 1); 204 received PT-Cy in combination with one IS drug—cyclosporine-A (CSA) or methotrexate (MTX) or mycophenolate-mofetil (MMF) (group 2), while 141 patients received PT-Cy in combination with two IS drugs—CSA + MTX or CSA + MMF (group 3). Transplants were performed from 2007 to 2015 and median follow-up was 20 months.

**Results:**

Probability of overall survival (OS) at 2 years was 50, 52.2, and 62.4%, for the three groups, respectively, *p* = 0.06. In multivariate analysis, in comparison to PT-Cy alone, the addition of two IS drugs was associated with reduced risk of extensive cGVHD (HR 0.25, *p* = 0.02). Use of bone marrow (BM) and anti-thymocyte globulin were independently associated with reduced risk of extensive cGVHD. Prognostic factors for non-relapse mortality (NRM) were the addition of two IS drugs to PT-Cy (HR 0.35, *p* = 0.04), diagnosis of AML, disease status at transplant, and patient CMV serology. Factors associated with increased OS were the use of PT-Cy with two IS drugs (HR 0.49, *p* = 0.02), AML, and disease status at transplant.

**Conclusion:**

For GVHD prophylaxis in MSD and UD HSCT, the addition of IS drugs to PT-Cy enhances its effect and reduces the risk of severe cGVHD, reducing mortality and improving survival.

## Background

Graft-versus-host disease (GVHD) remains one of the main life-threatening complications after allogeneic stem cell transplantation (HSCT) [1, 2]. The standard GVHD prophylaxis strategy is mostly based on the use of calcineurin inhibitors alone or in combination with other immunosuppressive (IS) drugs [3, 4]. This results in an incidence of 25–40% of acute GVHD and 40–60% of chronic GVHD after HSCT from HLA identical sibling (MSD) or unrelated donor (UD). The incidence of GVHD also depends on the conditioning regimen as well as patients and disease-related factors [5].

With the increased use of HSCT from unmanipulated haploidentical donor, adapted GVHD prophylaxis has been proposed [6, 7]. Among those, Luznik et al. [7] pioneered the use of high-dose post-transplant cyclophosphamide (PT-Cy) in combination with other IS drugs reporting a low incidence of acute (a) and chronic (c) GVHD and low transplant-related mortality.

The feasibility of PT-Cy in the haploidentical setting has prompted its use as sole GVHD prophylaxis in recipients of HSCT from MSD or UD [8, 9]. Luznik et al. [8] reported 43 and 10% of grades II–IV and III–IV aGVHD, respectively, and 10% of cGVHD in 117 patients receiving bone marrow (BM) transplantation from MSD with myeloablative regimen. Similar results were observed in a multicenter study [9].

However, the attempts to administer PT-Cy alone, in a phase 2 trial on adult patients with hematologic malignancies undergoing HSCT with peripheral blood stem cell (PBSC) grafts from either HLA identical sibling or unrelated donors, was associated with severe aGVHD and related deaths [10].

Mielcarek et al. [11] reported the use of PT-Cy followed by cyclosporine A (CSA), started on day + 5 post stem cell infusion, resulting in low incidence of severe aGVHD, but grade II–IV aGVHD and extensive cGVHD reached 77 and 30%, respectively.

More recently, the use of PT-Cy combined with other IS such as tacrolimus or mycophenolate mofetil (MMF) was shown to reduce the incidence of acute and chronic GVHD to 19 and 16%, respectively, in PBSC recipients [12].

We aimed to analyze PT-Cy alone or in combination with other IS as GVHD prophylaxis in a large cohort of patients transplanted for acute myeloid leukemia (AML) or acute lymphoblastic leukemia (ALL) and reported to the European Society for Blood and Marrow Transplantation (EBMT) Registry.

## Methods

### Study design

This is a retrospective registry-based analysis on behalf of the Acute Leukemia Working Party (ALWP) of the EBMT. The EBMT is a voluntary working group of more than 550 transplant centers that are required to report all consecutive stem cell transplantations and follow-up once a year. Audits are routinely performed to determine the accuracy of the data.

Adults (age > 18 years) with AML or ALL in complete remission (CR1 or CR2) or in advanced disease at transplant, reported to Promise-EBMT, who underwent a HSCT with MSD or 10/10 HLA matched UD using PT-Cy as first allogeneic HSCT between 2007 and 2015 were analyzed.

This study was approved by the ALWP of the EBMT institutional review board and conducted in accordance with the Declaration of Helsinki and Good Clinical Practice guidelines. All patients or legal guardians provided written informed consent authorizing the use of clinical information for research purposes.

A total of 423 patients were reported from 150 transplant centers, including 78 patients receiving PT-Cy alone (group 1) and 204 patients receiving PT-Cy in combination with one IS, mainly CSA or metothrexate (MTX) or MMF (group 2), while 141 patients received PT-Cy in combination with two IS drugs—CSA + MTX or CSA + MMF (group 3).

### End points and definitions

The primary end point was leukemia-free survival (LFS). Secondary end points were neutrophil engraftment, aGVHD and cGVHD, relapse incidence (RI), non-relapse mortality (NRM), GVHD-free relapse-free survival (GRFS), and overall survival (OS).

Neutrophil engraftment was defined as the first of 3 consecutive days with a neutrophil count of at least 0.5 × 109/L. Acute GVHD was graded according to the modified Seattle Glucksberg criteria [13] and cGVHD according to the revised Seattle criteria [14]. Relapse was defined as disease recurrence and appearance of blasts in the peripheral blood or BM (> 5%) after CR. NRM was defined as death from any cause other than relapse. Refined GRFS [15] was defined as survival without the following events: grade 3–4 acute GVHD, severe cGVHD, disease relapse, or death from any cause after transplantation. LFS was calculated until the date of first relapse, death from any cause, or the last follow-up for patients alive in CR.

Myeloablative conditioning (MAC) was defined as a regimen containing either total body irradiation (TBI) with a dose greater than 6 Gy, a total dose of oral busulfan (Bu) greater than 8 mg/kg, or a total dose of intravenous Bu greater than 6.4 mg/kg or melphalan at doses > 140 mg/m2. In addition, regimens containing two alkylating agents were also considered as MAC. All other regimens were defined as reduced intensity conditioning (RIC).

### Statistical analysis

Quantitative variables are described with median and range. Categorical variables are reported with counts and percent.

LFS and OS and GRFS were estimated by the Kaplan–Meier method. Cumulative incidence (CI) functions were used to estimate neutrophil engraftment, NRM, aGVHD, cGVHD, and RI. Competing risks were death for RI, relapse for NRM, and relapse or death for aGVHD and cGVHD. Univariate analyses were done using the log-rank test for GRFS, OS, and LFS, and Gray’s test for CI. For univariate analysis, comparisons were done using chi-square tests for categorical and Mann-Whitney tests for continuous variables. Multivariate analyses were performed using the Cox proportional hazard model.

There was no interaction between the different GVHD prophylaxis strategy and donor type; therefore, they were analyzed together.

Type of GVHD prophylaxis, diagnosis, disease status, age at transplant, transplant year, donor relatedness, stem cell source, cytomegalovirus (CMV) serostatus (donor and recipient negative vs. other combination), conditioning regimen, use of in vivo T cell depletion (anti-thymocyte globulin, ATG), and center experience were included in the final model. In order to test for center effect, we introduced a random effect or frailty for each center into the model [16].

The significance level was fixed at 0.05, and *p* values were two-sided. Statistical analyses were performed with the SPSS 22 (SPSS Inc./IBM, Armonk, NY, USA) and R 3.2.3 (R Development Core Team, Vienna, Austria) software packages.

## Results

### Patient and transplant characteristics

Table 1 summarizes the main characteristics by the GVHD prophylaxis strategies. Four hundred twenty-three patients were included in this study; most patients in both groups were transplanted for AML in CR1. The median follow-up was 20 months (95% CI 17.6–22.6). Patients in group 1 were younger (median age 37 years, *p* < 0.001) and transplanted in more recent years (2014, *p* < 0.001), received more frequently grafts from a MSD (80%, *p* < 0.001) and from a CMV-positive donor (73%, *p* = 0.008). In addition, group 1 received more often RIC (56%, *p* < 0.001) and BM as source of stem cells (74%, *p* < 0.001), with no ATG (100%, *p* < 0.001). Twenty-eight centers used ATG in combination with PT-Cy used, corresponding to 143 patients. Dose of ATG was available for 87 patients. Median dose for Thymoglobulin was 5 mg/kg (range 2.5–10 mg/kg). It was 50 mg/kg for ATG-Fresenius (range 20-60 mg/kg).

**Table 1:** Patient and disease characteristics

	PTCy alone (*n* = 78)	1 associated drug (*n* = 204)	2 associated drugs (*n* = 141)	*p* value
Median FU (median)	13.02 (10.13–15.9)	23.4 (20.15–26.7)	21.8 (15.42–28.12)	< 0.001
AGE at Tx, median (range) (IQR)	37.1 (18.1–73.7)(27.5–49.8)	51.3 (18.7–72.9)(37.9–60.2)	43.9 (18.1–76)(31.5–54.1)	< 0.001
Time diagnosis to Tx (months)	7.1 (2.1–81.8)(4.3–11.8)	5.3 (1.8–225.4)(3.7–10.8)	5.6 (0.4–186.9)(3.8–11.1)	0.137
Year of Tx, median (range)	2014 (2009–2015)	2013 (2008–2015)	2013 (2009–2015)	< 0.001
AML	56 (72%)	172 (84%)	103 (73%)	0.014
ALL	22 (28%)	32 (16%)	38 (27%)	
CR1	47 (60%)	135 (66%)	86 (61%)	0.366
CR2/3	8 (10%)	27 (13%)	24 (17%)	
Active disease	23 (30%)	42 (21%)	31 (22%)	
MSD	63 (81%)	114 (56%)	64 (45%)	< 0.001
UD	15 (19%)	90 (44%)	77 (54%)	
No F to M	61 (78%)	163 (82%)	106 (76%)	0.429
F to M	17 (22%)	37 (18%)	34 (24%)	
Missing	0	4	1	
KPS < 80	8 (11%)	13 (7%)	6 (4%)	0.189
KPS ≥ 80	64 (89%)	175 (93%)	130 (96%)	
Missing	6	16	5	
Pat. CMV negative	17 (23%)	68 (34%)	44 (32%)	0.218
Pat. CMV positive	57 (77%)	133 (66%)	92 (68%)	
Missing	4	3	5	
Donor CMV negative	19 (27%)	96 (48%)	55 (41%)	0.008
Donor CMV positive	51 (73%)	103 (52%)	80 (59%)	
Missing	8	5	6	
MAC	32 (44%)	96 (49%)	102 (74%)	< 0.001
RIC	41 (56%)	102 (51%)	36 (26%)	
Missing	5	6	3	
BM	58 (74%)	25 (12%)	25 (18%)	< 0.001
PB	20 (26%)	179 (88%)	116 (82%)	
No in vivo TCD	78 (100%)	130 (64%)	72 (51%)	< 0.001
In vivo TCD	0 (0%)	74 (36%)	69 (49%)	

*FU* follow-up, *CI* confidence interval, *IQR* interquartile range, *MAC* myeloablative, *RIC* reduced intensity conditioning regimen, *PTCy* post-transplant cyclophosphamide, *AML* acute myeloid leukemia, *ALL* acute lymphoblastic leukemia, *Tx* transplant, *UD* unrelated donor, *MSD* matched sibling donor, *CR* complete remission, *PB* peripheral blood, *BM* bone marrow, *TCD* T cell depletion, *CMV* cytomegalovirus, *KPS* Karnofsky performance status

### Neutrophil engraftment and GVHD

Patients receiving PT-Cy alone had 90% (95%CI 80.4–95.5) of neutrophil engraftment at 60 days, whereas it was 97% (95%CI 93.1–98.7) for patients in group 2 and 96.6% (95%CI 90.2–98.1) for group 3, *p* < 0.001. The median time to engraftment was longer for patients receiving PT-Cy alone (22 days), vs. 17.5 and 15 days for patients in groups 2 and 3, respectively.

CI of day 100 grade II–IV aGVHD and 1 year cGVHD were 27.9 and 33%, respectively.

In adjusted multivariate analysis (Table 2), there was no difference in the risk of grade II–IV aGVHD (group 1 vs. group 2 HR 0.63, 95%CI 0.28–1.3, *p* = 0.23; group 1 vs. group 3 HR 1.4, 95%CI 0.65–3.14, *p* = 0.38) and grade III–IV aGVHD (group 1 vs. group 2 HR 0.52, 95%CI 0.17–1.5, *p* = 0.25; group 1 vs. group 3 HR 0.81, 95%CI 0.27–2.44, *p* = 0.71) according to the groups.

**Table 2:** Multivariate analysis

	HR	95% CI	*p* value
OS			
PTCy alone	Reference		
PT-Cy + 1 drug	0.72	0.40–1.30	0.27
PT-Cy + 2 drugs	0.49	0.26–0.93	0.03
Age (per 10 years)	1.11	0.97–1.28	0.18
AML vs. ALL	0.60	0.39–0.91	0.02
Year of Tx	0.99	0.88–1.12	0.90
UD vs. MSD	0.95	0.66–1.36	0.77
Disease status			
CR1	Reference		
CR2/C3	1.7	1.07–2.70	0.02
Active disease	2.48	1.71–3.59	< 0.001
RIC vs. MAC	1.20	0.83–1.74	0.34
PB vs. BM	1.13	0.69–1.83	0.63
In vivo TCD	1.07	0.72–1.60	0.73
Patient positive CMV serology	1.07	0.73–1.58	0.73
Donor positive CMV serology	1.17	0.81–1.71	0.40
Center (frailty)			0.25
GRFS			
PTCy alone	Reference		
PT-Cy + 1 drug	0.72	0.45–1.16	0.18
PT-Cy + 2 drugs	0.51	0.31–0.84	0.007
Age (per 10 years)	0.96	0.87–1.07	0.48
AML vs. ALL	0.89	0.62–1.26	0.50
Year of Tx	0.97	0.88–1.08	0.60
UD vs. MSD	1.04	0.78–1.40	0.77
Disease status			
CR1	Reference		
CR2/C3	1.27	0.85–1.89	0.24
Active disease	2.18	1.61–2.95	< 0.001
RIC vs. MAC	1.32	0.98–1.77	0.07
PB vs. BM	1.30	0.87–1.94	0.20
In vivo TCD	0.83	0.60–1.16	0.27
Patient positive CMV serology	0.95	0.69–1.31	0.77
Donor positive CMV serology	1.20	0.88–1.63	0.26
Center (frailty)			0.91
LFS			
PTCy alone	Reference		
PT-Cy + 1 drug	0.97	0.57–1.63	0.90
PT-Cy + 2 drugs	0.65	0.37–1.12	0.12
Age (per 10 years)	1.09	0.97–1.23	0.17
AML vs. ALL	0.68	0.46–0.99	0.04
Year of Tx	1	0.89–1.11	0.94
UD vs. MSD	0.92	0.659–1.27	0.60
Disease status			
CR1	Reference		
CR2/C3	1.64	1.07–2.51	0.02
Active disease	2.49	1.78–3.47	< 0.001
RIC vs. MAC	1.255	0.9–1.75	0.18
PB vs. BM	0.94	0.61–1.45	0.78
In vivo TCD	1.07	0.75–1.54	0.70
Patient positive CMV serology	0.98	0.69–1.39	0.93
Donor positive CMV serology	1.24	0.89–1.74	0.21
Center (frailty)			0.91
Acute GvHD II-IV			
PTCy alone	Reference		
PT-Cy + 1 drug	0.62	0.29–1.36	0.23
PT-Cy + 2 drugs	1.40	0.65–3.01	0.38
Age (per 10 years)	1.01	0.87–1.18	0.90
AML vs. ALL	0.53	0.33–0.85	0.008
Year of Tx	0.91	0.80–1.04	0.16
UD vs. MSD	1.66	1.06–2.60	0.03
Disease status			
CR1	Reference		
CR2/C3	0.62	0.32–1.19	0.15
Active disease	1.14	0.71–1.84	0.59
RIC vs. MAC	1.72	1.10–2.70	0.02
PB vs. BM	1.59	0.83–3.02	0.16
In vivo TCD	1.18	0.74–1.89	0.48
Patient positive CMV serology	0.74	0.47–1.15	0.18
Donor positive CMV serology	1.78	1.13–2.79	0.01
Center (frailty)			0.23
Chronic GvHD			
PTCy alone	Reference		
PT-Cy + 1 drug	0.60	0.27–1.37	0.23
PT-Cy + 2 drugs	0.52	0.22–1.23	0.14
Age (per 10 years)	0.90	0.76–1.05	0.18
AML vs. ALL	1.01	0.58–1.75	0.97
Year of Tx	0.97	0.82–1.14	0.69
UD vs. MSD	0.99	0.64–1.54	0.96
Disease status			
CR1	Reference		
CR2/C3	0.52	0.24–1.09	0.08
Active disease	0.84	0.47–1.48	0.54
RIC vs. MAC	1.01	0.63–1.62	0.96
PB vs. BM	2.41	1.19–4.89	0.01
In vivo TCD	0.59	0.37–0.96	0.03
Patient positive CMV serology	0.88	0.55–1.43	0.61
Donor positive CMV serology	0.86	0.53–1.39	0.53
Center (frailty)			0.93
Extensive chronic GvHD			
PTCy alone	Reference		
PT-Cy + 1 drug	0.57	0.21–1.56	0.27
PT-Cy + 2 drugs	0.25	0.08–0.84	0.02
Age (per 10 years)	0.76	0.63–0.96	0.02
AML vs. ALL	1.64	0.65–4.16	0.30
Year of Tx	1	0.79–1.26	0.98
UD vs. MSD	1.26	0.68–2.33	0.47
Disease status			
CR1	Reference		
CR2/C3	0.56	0.19–1.63	0.29
Active disease	1.24	0.57–2.68	0.59
RIC vs. MAC	1.43	0.72–2.84	0.30
PB vs. BM	4.57	1.61–12.99	0.004
In vivo TCD	0.23	0.01–0.54	< 0.001
Patient positive CMV serology	0.92	0.46–1.80	0.80
Donor positive CMV serology	0.76	0.38–1.55	0.45
Center (frailty)			0.94
Relapse			
PTCy alone	Reference		
PT-Cy + 1 drug	1.12	0.59–2.13	0.73
PT-Cy + 2 drugs	0.82	0.42–1.61	0.57
Age (per 10 years)	1.03	0.89–1.19	0.69
AML vs. ALL	0.97	0.59–1.6	0.91
Year of Tx	1.03	0.90–1.18	0.64
UD vs. MSD	0.87	0.58–1.30	0.50
Disease status			
CR1	Reference		
CR2/C3	1.64	0.97–2.80	0.07
Active disease	2.42	1.60–3.66	< 0.001
RIC vs. MAC	1.18	0.78–1.77	0.44
PB vs. BM	0.83	0.50–1.38	0.47
In vivo TCD	0.89	0.57–1.39	0.61
Patient positive CMV serology	0.71	0.46–1.08	0.11
Donor positive CMV serology	1.34	0.88–2.04	0.17
Center (frailty)			0.92
NRM			
PTCy alone	Reference		
PT-Cy + 1 drug	0.69	0.27–1.73	0.42
PT-Cy + 2 drugs	0.358	0.13–0.99	0.05
Age (per 10 years)	1.195	0.96–1.48	0.11
AML vs. ALL	0.353	0.19–0.67	0.001
Year of Tx	0.95	0.78–1.15	0.60
UD vs. MSD	1.022	0.58–1.81	0.94
Disease status			
CR1	Reference		
CR2/C3	1.63	0.78–3.38	0.19
Active disease	2.949	1.65–5.27	< 0.001
RIC vs. MAC	1.339	0.74–2.42	0.33
PB vs. BM	1.247	0.56–2.78	0.59
In vivo TCD	1.47	0.78–2.75	0.23
Patient positive CMV serology	2.042	1.03–4.04	0.04
Donor positive CMV serology	1.038	0.58–1.87	0.90
Center (frailty)			0.29

*HR* hazard ratio, *CI* confidence interval, *aGVHD* acute graft-versus-host disease, *cGVHD* chronic GVHD, *NRM* non-relapse mortality, *OS* overall survival, *LFS* leukemia-free survival, *GRFS* GvHD-free relapse-free survival, *MAC* myeloablative, *RIC* reduced intensity conditioning regimen, *PTCy* post-transplant cyclophosphamide, *AML* acute myeloid leukemia, *ALL* acute lymphoblastic leukemia, *Tx* transplant, *UD* unrelated donor, *MSD* matched sibling donor, *CR* complete remission, *PB* peripheral blood, *BM* bone marrow, *TCD* T cell depletion, *CMV* cytomegalovirus

Diagnosis of ALL (HR 0.57, 95%CI 0.32–0.84, *p* < 0.001), UD (HR 1.65, 95%CI 1.1–2.6, *p* = 0.02), RIC regimen (HR 1.72, 95%CI 1.1–2.7, *p* = 0.01), and donor CMV positive (HR 1.77, 95%CI 1.1–2.8, p = 0.01) were independently associated with increased risk of grade II–IV aGVHD.

**Fig. 1:**
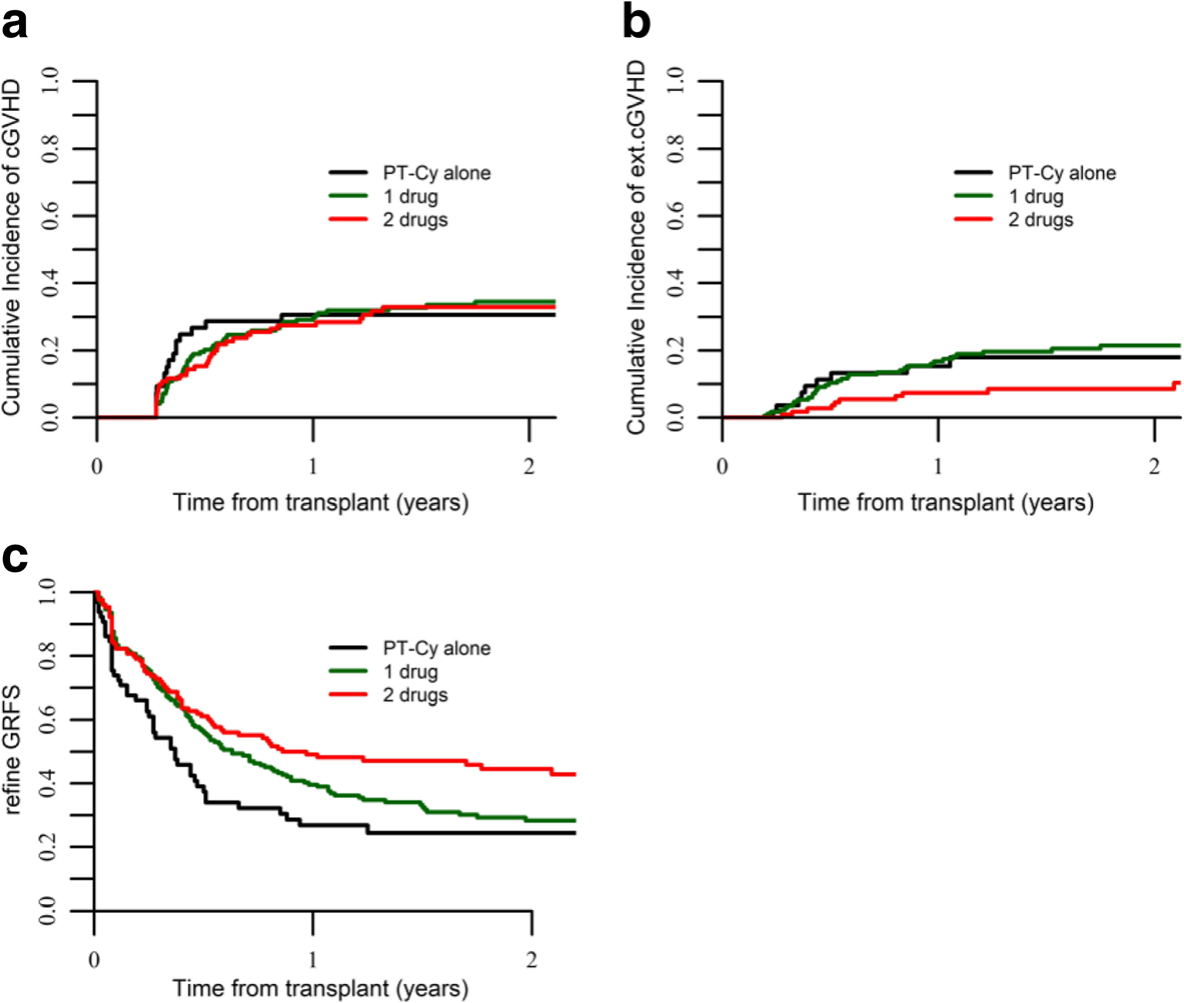
**a** cGVHD, **b** extensive cGVHD, and **c** GRFS by GVHD prophylaxis strategy

The type of GVHD prophylaxis did not impact the CI of cGVHD (31, 34, and 33% *p* = 0.92, respectively) (Fig. 1a). Similarly, the intensity of the GVHD prophylaxis was not associated with cGVHD (group 1 vs. group 2 HR 0.60, 95%CI 0.26–1.36, *p* = 0.22; group 1 vs. group 3 HR 0.54, 95%CI 0.22–1.23, *p* = 0.13) in the multivariate analysis (Table 2).

The use of BM (HR 0.41, 95%CI 0.28–0.48, *p* = 0.01) and the absence of ATG (HR 0.59, 95%CI 0.36–0.94, *p* = 0.03) were independently associated with a reduced risk of cGVHD.

The incidence of extensive cGVHD was higher for patients receiving PT-Cy alone (18%) or PT-Cy + 1 IS (20%) vs. 8.5% for those having PT-Cy + 2 IS (Fig. 1b). This was also confirmed in the multivariate analysis (Table 2) where in comparison to PT-Cy the addition of two IS was associated with reduced risk of extensive cGVHD (group 1 vs. group 2, HR 0.56, 95%CI 0.20–1.5, *p* = 0.27; group 1 vs. group 3, HR 0.25, 95%CI 0.07–0.84, *p* = 0.02). The use of BM vs. PBSC grafts (HR 0.21, 95%CI 0.07–0.62, *p* < 0.001) and the absence of ATG (HR 0.22, 95%CI 0.09–0.54, *p* < 0.001) were also independently associated with reduced risk of extensive cGVHD.

### Relapse and NRM

**Fig. 2:**
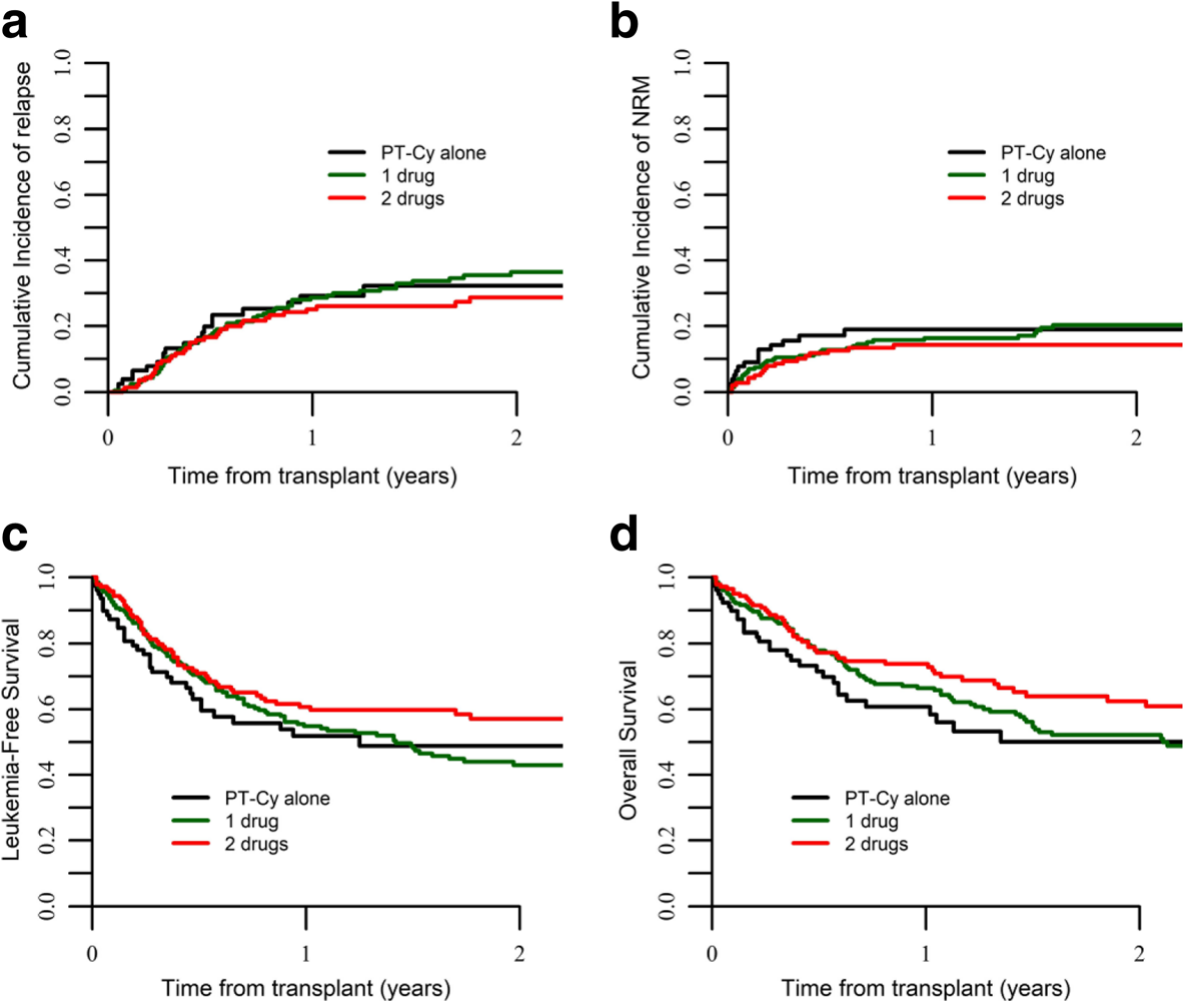
**a** RI, **b** NRM, **c** LFS, and **d** OS by GVHD prophylaxis strategy

At 2 years, the CI of relapse for the whole cohort was 33% with no difference according to groups (32 vs. 36 vs. 28%, *p* = 0.47) (Fig. 2a). CI of relapse was 33.9% for AML and 32.1% for ALL, *p* = 0.04, and it was 27.4, 38.5, and 47% for patients transplanted in CR1, CR2, and in advanced disease status *p* < 0.01, respectively. According to donor type, CI of relapse was 37.5 and 28.5% for MSD and UD, *p* = 0.21, respectively.

Overall, 2 years NRM was 18% with no difference for the three groups (19 vs. 20 vs. 14%, *p* = 0.47) (Fig. 2b). Overall, main causes of death were disease recurrence (50%), infections (17%), and GVHD (15%).

In multivariate analysis (Table 2), the type of GVHD prophylaxis was not associated with relapse, with advanced disease status accounting for an increased risk of relapse (HR 2.42, 95%CI 1.60–3.66, *p* < 0.001).

As for NRM (Table 2), the addition of two IS to the PT-Cy (group 1 vs. group 3) (HR 0.35, 95%CI 0.12–0.91, *p* = 0.04), the diagnosis of AML (HR 0.35, 95%CI 0.18–0.66, *p* = 0.001), advanced disease status at transplant (HR 2.94, 95%CI 1.65–5.29, *p* < 0.001), and patient CMV positive serology (HR 2.04, 95%CI 1.30–4.04, *p* = 0.04) were independently associated with the risk of NRM.

### OS, LFS, and GRFS

OS, LFS, and GRFS at 2 years were 55, 48, and 33%, respectively. According to GVHD prophylaxis, OS was 50 vs. 52 vs. 62%, *p* = 0.06; LFS was 49% vs. 43% vs. 57%, *p* = 0.08; and GRFS was 24% vs. 28% vs. 44%, *p* < 0.001, for patients receiving PT-Cy alone or PT-Cy + 1 IS or PT-Cy + 2 IS, respectively (Figs. 1c, 2c, d).

OS was 53% for patients transplanted from a MSD and 77% for those transplanted from a UD (*p* = 0.56), and it was 57 and 49% (*p* = 0.40) for AML vs. ALL, respectively.

In multivariate analysis (Table 2), factors associated with superior OS were the use of PT-Cy in combination with two IS (group 1 vs. group 3; HR 0.49, 95%CI 0.26–0.93, *p* = 0.02), diagnosis of AML (HR 0.59, 95%CI 0.39–0.90, *p* = 0.001), and disease status at transplant (CR1 vs. CR2 HR 0.58, 95%CI 0.37–0.93, *p* = 0.0; CR1 vs. advanced HR 0.40, 95%CI 0.27–0.58 *p* < 0.001). Of note, the intensity of the GVHD prophylaxis had an impact on GRFS (group 1 vs. group 3; HR 0.51, 95%CI 0.31–0.83 *p* < 0.001) (Table 2).

## Discussion

In this study, we compared the efficacy of PT-Cy GVHD prophylaxis given alone or in combination with one or two other immunosuppressive drugs in a homogenous group of adult patients with acute leukemia undergoing HSCT from MSD or 10/10 UD. We observed significant differences in the incidence of severe cGVHD and mortality in correlation with the intensity of the GVHD prophylaxis with the combination of PT-Cy plus two IS drugs (either CSA-MTX or CSA MMF), resulting in improved survival.

The biological mechanism by which PT-Cy aids in preventing GVHD after BM graft has been previously described and involves in vivo selective destruction of alloreactive T cells, induction of tolerance, and intra-thymic clonal deletion of alloreactive T lymphocytes [17].

The use of PT-Cy alone, without the use of any additional IS drugs, for GVHD prophylaxis in the setting of matched sibling donor or 10/10 HLA matched unrelated donor was initially reported by Luznik [8] and, subsequently, in a multicenter study, [18] both demonstrating the efficacy of this strategy. Importantly, in these studies, BM was the sole stem cell source. Notably, these findings have not been demonstrated with PBSC [19, 20]. Alousi [19] recently published the results of a phase II clinical trial using PBSC and RIC regimen indicating an excess of acute and chronic GVHD and NRM and therefore recommending the use of the standard GVHD prophylaxis in HSCT following RIC regimen in combination with PBSC grafts. The same findings determined the early closure of a different prospective phase 2 trial after four cases of severe acute GVHD, and related toxicity was reported on the first five patients enrolled [20].

Similarly, an increased risk of GVHD has been reported in haploidentical HSCT with PT-Cy with PBSC grafts [21]. One may argue that the higher number of CD3+ cells in the PBSC grafts could be in part responsible for these results. Importantly, in our study, the use of PBSC as stem cell source was significantly associated with the increased risk of cGVHD in the multivariate analysis, and the effect of the intensity of the GVHD prophylaxis remained independently associated to the risk of severe cGVHD, mortality, and survival.

In view of the high incidence of GVHD with PT-Cy as single agent, especially in PBSC recipients, it seems that PT-Cy should be combined with additional IS. Nevertheless, there is still not enough data to determine the most effective IS drug to be used in association with PT-Cy.

In an attempt to reduce the risk of severe GVHD, some authors [11] added CSA starting on day + 5 after PT-Cy infusion in 43 patients with hematological diseases receiving PBSC from MSD or UD. Although a lower cumulative incidence of cGVHD and no grade III–IV aGVHD were reported, the high incidence of grade II aGVHD over 70% highlights the importance of optimization of the PT-Cy GVHD prevention regimen, in order to reduce GVHD incidence and the potential related toxicity and mortality.

In HLA identical related and unrelated grafts, the addition of MMF and tacrolimus to PT-Cy allowed satisfactory control of acute GVHD (ranging 17%, with no grade IV) resulting in very low NRM (3% at 2 years) in a previous study [22]. Currently, a clinical trial evaluating this GVHD prevention combination is ongoing in allogeneic stem cell transplantation recipients, following a MAC or RIC preparative regimen (NCT03128359). In our study, we are not able to evaluate this GVHD prophylaxis due to the unbalanced distribution of this specific drug combination in our cohort (exclusively used in UD recipients and mainly in a single center). The question of the optimal combination remains a matter of debate not just in HSCT from HLA matched siblings and unrelated donors but, also, in the haploidentical setting at least until prospective comparison study results are not available.

We are aware that in our study there may be unmeasured factors that have not been considered, and this is a limitation when conducting retrospective studies. The impact of ATG could not be studied thoroughly because of the absence of ATG in the patients receiving PT-Cy as the sole anti-GVHD prophylaxis. We cannot discard the possibility that this could have played a role in the observed increased incidence of graft failure and extensive cGVHD. The protective effect of ATG in reducing cGVHD and improving GRFS has been recently demonstrated in a large phase clinical III trial [23] on transplantation of peripheral blood stem cells from HLA identical siblings and the myeloablative conditioning regimen. It is possible that the addition of ATG to PT-Cy may reduce GVHD incidence. However, the best timing and dose of ATG in combination with PT-Cy should be further evaluated in clinical trials.

One of the limitations of our registry-based study, including patients in all disease status, is that some disease characteristics could be confounding factors. In order to overcome this limitation, we performed a subgroup analysis in a homogenous group of patients with AML in CR1 and not receiving ATG. Despite the low number of patients, the results are consistent with those in the entire population, but the low numbers in each subgroup prevent to achieve enough statistical power. In our study for patients who received PT-Cy in combination with two other IS drugs, the benefit of adding immunosuppressive drugs to PT-Cy was observed regardless of the use of ATG (data not shown).

The idea of sparing long-term immunosuppression by using PT-Cy in patients with high-risk leukemia [24] and thus reducing relapse rates, especially in the early post-transplant period, is attractive and deserves further investigation. Importantly, in our study, the intensity of the GVHD prophylaxis did not modify the risk of relapse. This led to an advantage in OS and, importantly, in GRFS, which reflects the quality of life without long-term complications related to the GVHD.

In our cohort, disease status at HSCT remained the only factor associated with increased relapse, highlighting the importance of reducing the disease burden before HSCT. In this context, some authors [25] showed how Cy in the early post-transplant period is responsible for a selective depletion of alloreactive T cells while sparing those mediating the graft vs. leukemia (GVL). Also recently, another group [26] reported the complete abrogation of the proliferation of donor-derived natural killer (NK) cells 8 days following PT-Cy infusion in haplo-recipients, with donor NK cells expressing less mature phenotype NKG2A and CD26L and with slow reconstitution of recipient NK cells thereafter. The mechanism of NK cell reconstitution in the setting of MSD and UD with PT-Cy needs to be further evaluated.

Given our results, the use of additional immunosuppressive drugs along with PT-Cy in matched sibling or unrelated donor transplants is effective, reducing the risk of graft failure and severe chronic GVHD and improving overall survival. This strategy of GVHD prophylaxis could be an important tool for the post-transplant immunomodulation, also in the setting of mismatched unrelated donor transplants, and could represent a platform for early withdrawal of the immunosuppression enhancing the GVL in high-risk leukemic patients.
